# Use of pager devices in New Zealand public hospitals as a critical communication tool: Barriers & way forward

**DOI:** 10.1016/j.heliyon.2023.e18717

**Published:** 2023-07-26

**Authors:** Ehsan Ullah, Mirza Mansoor Baig, Hamid GholamHosseini, Jun Lu

**Affiliations:** a Auckland District Health Board, Auckland, New Zealand; b Data Science Team, Orion Health, Auckland, New Zealand; c School of Engineering, Computer and Mathematical Sciences, Auckland University of Technology, Auckland, 1142, New Zealand; d Auckland Bioengineering Institute, University of Auckland, New Zealand; e Maurice Wilkins Centre for Molecular Discovery, Auckland, 1010, New Zealand; f College of Food Science and Technology, Zhejiang University of Technology, Hangzhou, China; g College of Food Engineering and Nutrition Sciences, Shaanxi Normal University, Xi'an, China

**Keywords:** Pager devices, Paging system, In-hospital communication, Healthcare technology

## Abstract

**Objectives:**

The aim of this study was to analyse the current use, identify challenges and barriers and propose a way forward for the use of the pager devices in the in-hospital communications.

**Methods:**

Initially, 447 studies were identified through database searching. After checking against the eligibility, 39 studies were included. Full-text records were retrieved and reviewed by two authors. After excluding unrelated studies and duplicate records, a total of 12 articles were selected for the final review.

**Results:**

The use of pagers often lacks standardisation, content, format, urgency level, and clarity within the message. Some studies reported that medical staff preferred in-person interactions with consults instead of communicating over the phone or pagers. Productive communication can reduce the turnaround time by up to 50%. The key challenges are; (1) data security and privacy, (2) timely acknowledgement of received communication, (3) lack of two-way communications causing issues in critical care situations and (4) there is no standard process for the in-hospital communications.

**Conclusion:**

We found that the clinicians’ age, experience, speciality and preferences greatly matter and influence the selection of tools and technology in healthcare. With revolutionary advances in technology, smartphones have inevitably become beneficial to healthcare, owing to multiple instant messaging applications (apps) that can streamline encrypted clinical communication between medical teams and could be safely used for in-hospital communications.

## 1 Introduction

Pager devices have been used for several decades across the globe. They have allowed for increased information transmission between hospital teams and individuals. They have served as the main communication tool between the nursing staff at the bedside and the medical staff who are responsible for providing advice when required. Even though pagers are still considered the primary tool for such communications, some medical staff believe that pagers are often disruptive [1]. Multiple reports have highlighted that the proportion of urgent and semi-urgent pager alerts during an on-call shift for resident medical officers (RMOs) led to a significant increase in their workload and was later determined to be non-urgent [[2], [3], [4]]. Furthermore, as the load of pager calls increases, the on-call RMOs become unable to respond, and the purpose for which the pager system exists starts to fail [5]. Although a direct cause and effect relationship has not been established yet, there seems to be a clear correlation between increased workload and an overall adverse effect on patient safety [1,3].

This study aimed to analyse the current use of pagers in-hospital for communications. Also, we identified challenges with the use of the pager devices in in-hospital communications and opportunities available as alternatives.

## 2 Background

The alphanumeric paging system or pager devices were introduced into healthcare in the 1950s as a one-way communication tool using a numeric code and then quickly evolved to include alphabetic text-based messaging [3]. Pager devices provide wireless communication between hospital teams and individual staff members. Taranaki District Health Board (and many other District Health Boards within New Zealand) use Pager devices (Alphanumeric Pager, model number W2028P) supplied by Wex International Limited, a Hong Kong-based company specialising in wireless, radiofrequency, GPS/GPRS and Pager technology. The model used by New Zealand hospitals operates at 138–174, 138–174, 278–284, 405–480, 928–932 MHz Bands and channel spacing of 25 KHz. It uses Post Office Code Standardisation Advisory Group (POCSAG) signal format and can transmit data at 512, 1200 or 2400 bits per second (bps). The maximum alphanumeric characters in one pager message are 120, and it can display 20 characters per line and has a maximum of four-line display. It can store up to 16 messages.

Pagers have four different alert tones, continuous audible alert, standard alert tone for routine pager messages, emergency alert with the fixed alert tone, and vibration. A pager device requires one AAA (1.5 V) battery and has a battery backup life of 15 s (when the battery is replaced, a longer delay means messages will be lost). Battery life varies from 1600 h (2400 bps) to 2000 h (512 bps). The W2028P model weighs 135 g (including battery) and has a size of 80.5L x 54.2W × 18.60H mm without the belt clip. The pager displays time and date, indicating if the pager is within reception range and its battery status.

## 3 Methodology

### 3.1 Articles search, selection criteria and results

In order to review the literature that deals with the use of pager devices for communications in hospital settings, related keywords were used to cross-search for thousands of related papers in significant databases of scientific publications, including IEEE Xplore, Springer Link, Scopus and PubMed Library.

We chose the preferred reporting items for systematic reviews and meta-analyses (PRISMA) as the systematic review methodology [6]. One of the authors conducted an initial screening of the retrieved records. Duplicated articles were eliminated, and additional records were excluded after reviewing individual titles and abstracts. A second author then reviewed the included studies and evaluated the full-text articles or eligibility.

The eligibility criteria for inclusion in the review are.

1. Original articles mainly published as a journal article
2. Paper published or reported between 2015 and 2022 (inclusive)
3. The use of pager devices for communication in the patient care processes in the hospital settings was the primary subject of this study
4. Written and published in English
5. Keywords: ‘use of pager in hospital’, ‘hospital communications tools’, ‘paging system’, 'in-hospital communication’ and ‘challenges and issues with pager system in hospital’

We excluded articles that were not considered original research, such as letters to editors, opinions, position papers, commentaries or review articles. Initially, 347 studies were identified through database searching for the keywords enlisted above. A total of 308 records did not meet our inclusion criteria based on the initial screening, and therefore, 39 studies were included for checking against the eligibility. Full-text papers were retrieved and reviewed by two authors for completeness and quality. The completeness of the included studies was assessed by the availability of the full-text articles inlcuding results or findings which were coherent to the study design and methodology. The quality of the included studies was assessed by checking the authenticity of the publication in a peer reviewed journal, availability of the authors’ affiliations and contact details of the corresponding authors. After excluding unrelated studies and duplicate records, 12 articles were deemed suitable for selection, and for the final review by all authors to identify the characteristics of pager devices, to understand the pros and cons of using pager devices for hospital communication, and to assess the privacy and secruity of information that is communicated through these devices.

## 4 Analysis of the use of pager in hospital communications

### 4.1 One-way communication in hospital

Cellular phones and smartphones have embarked widely in the last couple of decades. This has created numerous options for rapid communication to overcome the limitations of the alphanumeric paging system (such as the limit of text and display), allowing transmission of rich textual and graphic data in a bi-directional fashion [7]. These innovations have increased the speed of information transfer and enabled other essential functions, such as removing the requirement to wait for a reply as with pager devices. The communication between attending nursing staff or family members and secondary responders about a patient's clinical deterioration is often quite complex. The current set of communication tools i.e. telephone and particularly pager devices, and procedures i.e. ‘777-call’ and ‘paging’ while serve the purpose yet not ideal. It is of paramount significance to have bi-directional communication between attending nursing staff and secondary responders. The richness of text and graphic data shared between these groups of staff in real-time through smartphone app would mean improved assessment of the situation by secondary responders, and faster response. These characteristics of smartphone technology would enable better team worker between various components of rapid response teams (RRTs) and promise better patient outcomes [8]. However, these apparent benefits of the smartphone use for communication of healthcare information between teams do come with unintended consequences [[9], [10], [11], [12]].

A study by Fiorinelli et al. [9] investigated the nurse distraction with using modern mobile technology and found that although the use of current tools and technologies can improve nurses' performance and the quality of care provided. However, the application of regulations and policies by healthcare facilities is desirable to avoid inappropriate use of these devices by nurses. The available data do not provide a precise estimate of the effect that distraction from smartphones has on the outcomes of nursing care [9].

### 4.2 Challenges and barriers for using pager devices in hospital communications

Studies conducted within the last ten to twelve years have shown that the use of smartphones is associated with reduced volume and quality of face-to-face interactions between healthcare providers such as nurses and patients [[13], [14], [15]]. Smartphones have also been blamed for limiting the opportunities for more meaningful interactions between healthcare professionals and teams, despite the perception that they have reduced the time required to reach out to someone such as a senior clinician [13,16].

From a technical standpoint, cellular network reliability, unpredictable cellular receptivity within certain areas of the hospital ‘dead zones’ [17], such as thick-walled rooms (radiology, oncology therapy and other equipment rooms have lead-lined walls). Another major barrier to the implementation of cellular smartphones as a primary tool for the communication within healthcare is the data privacy concerns, as there is no certainty about the security of the data and there is a risk of losing the confidentiality of the information [[18], [19], [20]].

In New Zealand, the Privacy Act 2020 [21] stresses more strongly on data security than ever before. The author has also reviewed the privacy management of new data sources, including smartphone applications for healthcare and found that the risk of loss of confidentiality of the healthcare data could be breached at multiple levels [22]. Another consideration would be cost involved as the healthcare organisations would need to provide smartphones with a higher level of data security and identification authentication features to its staff, which is considerably more expensive than pager devices. The unintended consequences of smartphone use within healthcare context explained above, and the technical issues mentioned in the above paragraph must be addressed before healthcare communications can fully benefit from the advancement brought out by smartphones. The smartphones could only replace the pagers as a primary tool for communication within healthcare.

### 4.3 Privacy and security

Security and privacy in healthcare are critical. Overall, security is a concept similar to the safety of any system. As the transmission of data in any communication application is wireless, it may result in various security threats. Such threats have the potential to pose serious problems to an individual's social life. Security issues in wireless sensor networks are a significant area of research in recent years, and many researchers have specifically addressed security issues with respect to healthcare applications [9,10,[23], [24], [25]].

Security issues can be classified into system security and information security. Ng et al. [26] have classified threats and attacks into two major categories—passive and active. Kargl et al. [27] have mentioned attacks in health monitoring in detail such as; modification of medical data, forging of alarms on medical data, denial of service, location and activity tracking of users, physical tampering with devices and jamming attacks. Security and privacy vulnerabilities are discussed in detail by Williams [28]. Some of the key points mentioned are; ease of network formation, the complexity of interactions, duplicitous users, and leakage to third party servers and shared content.

## 5 Discussion

On average, medical staff are paged 3.5 times per hour and spend 7% of their time returning pages, 8% of these pages are from other physicians, and 80% are from nurses [29]. These pages often lack standardization, content, format, urgency level, and clarity within the message. Some studies reported that the medical staff were found to prefer in-person interactions with consults instead of over-the-phone consults or pagers due to a higher level of productive communication, reducing turnaround time by up to 50% [[29], [30], [31]].

We found that the medical staff prefer utilising technology to improve efficiency and communications, ranging from pagers to more sophisticated digital applications. There was no significant difference in age when accounting for rounding preferences that involved technology or modern apps [3,32]. Clinicians also prefer to communicate with the consultant via text or phone more often than face-to-face talk. Unfortunately, while clinicians have technology and resources available, they do not always use them or are not suitable or customised to their use [30,31,33,34].

This study struggled to find any current information on what is considered to be a ‘gold standard’ for in-hospital communications. In the absence of a recognised process, a collection of study findings can serve a leader well when designing the communication process. One of the study suggested that the hospitals should use text messaging or phone applications over physical pagers [9,33,35,36]. Since 80% of pages come from nurses, it is imperative that nurses and other staff responsible for sending pages be properly trained to use standardized templates. In analysing the process design concepts of one-way communications, general preferences may be established, standardized physician responding preferences to improve efficiency based on the two categories of work assignment and communication [[29], [30], [31],^33^,^34^].

The concept of communication has expanded globally, including details on the transmitter, receiver, acknowledgement, actions, tasks and more. All of these are governed by national and international data privacy and data protection laws such as Health Insurance Portability and Accountability Act (HIPAA) and General Data Protection Regulation (GDPR) [23,24,37].

## 6 Conclusion

Effective patient care is vital for efficient clinical communication between teams in healthcare settings. With miscommunication being recognised as one of the most common causes of preventable patient morbidity and mortality, choosing the most efficient route for the prompt retrieval and transfer of messages has become a critical factor in preventing adverse patient outcomes. The mid-20th century saw a breakthrough in intra-hospital communication with the introduction of alphanumeric pagers. To this day, pagers remain the primary method of communication in hospitals around the world, used for effective communication within a certain team or speciality. However, they are a predominantly one-way communication method, as one needs a telephone to pager or reply. It can also take more than a few minutes to send and receive a response [12,35,38].

In 2014, a study carried out at Stanford University Hospital labelled the paging system as inefficient and unreliable, as it was not compliant with the 1996 HIPAA, which was created primarily to modernise the flow of healthcare information and stipulate how personal information maintained by the healthcare and healthcare insurance industries should be protected from fraud and theft as regulated by the Centers for Disease Control and Prevention (CDC) [10,11,34,36].

This study found that there is no standard process established for in-hospital communications. The current process has a significant variation between the specialities and found a huge difference in medical staff preferences when selecting tools and technology. This study noted that the clinicians’ age, experience, speciality, and preference greatly matter and influence the critical communication process or strategy for in-hospital communications. Moreover, today with revolutionary advances in technology, smartphones have become beneficial to healthcare due to multiple instant messaging applications (apps) that can streamline encrypted clinical communication between medical teams and safely be used for in-hospital communications.

## Ethics approval

Not Applicable.

## Author contribution statement

All authors listed have significantly contributed to the development and the writing of this article.

## Data availability statement

Data included in article/supp. material/referenced in article.

## Additional information

Editor Paper: APC waived - waiver code: Heliyon - Contractual payment discounts.

## Declaration of competing interest

The authors declare that they have no known competing financial interests or personal relationships that could have appeared to influence the work reported in this paper.
